# Transcriptomics-Based Approach Identifies Spinosad-Associated Targets in the Colorado Potato Beetle, *Leptinotarsa decemlineata*

**DOI:** 10.3390/insects11110820

**Published:** 2020-11-21

**Authors:** Pierre Bastarache, Gabriel Wajnberg, Pascal Dumas, Simi Chacko, Jacynthe Lacroix, Nicolas Crapoulet, Chandra E. Moffat, Pier Morin

**Affiliations:** 1 Department of Chemistry and Biochemistry, Université de Moncton, 18 Antonine-Maillet Avenue, Moncton, NB E1A 3E9, Canada; epb6696@umoncton.ca (P.B.); epd3362@umoncton.ca (P.D.); 2 Atlantic Cancer Research Institute, Pavillon Hôtel-Dieu 35 Providence Street, Moncton, NB E1C 8X3, Canada; Gabrielw@canceratl.ca (G.W.); simic@canceratl.ca (S.C.); jacynthe.lacroix@canceratl.ca (J.L.); nicolas.crapoulet@vitalitenb.ca (N.C.); 3 Fredericton Research and Development Centre, Agriculture and Agri-Food Canada, 850 Lincoln Road, Fredericton, NB E3B 4Z7, Canada; chandra.moffat@canada.ca

**Keywords:** Colorado potato beetle, insecticides, microRNAs, next-generation sequencing, non-coding RNAs, spinosad, transcriptomics

## Abstract

**Simple Summary:**

The Colorado potato beetle *Leptinotarsa decemlineata* is a potato pest that can cause substantial damages to potato crops worldwide. Multiple approaches have been leveraged to control this pest including the use of a variety of insecticides. Resistance to different insecticides aimed at controlling this insect has been reported and much work has been conducted in recent years to elucidate the underlying molecular changes associated with insecticide resistance in *L. decemlineata*. However, information is sparse regarding the molecular impact associated with spinosad treatment in this insect pest. The current study thus explores transcriptional changes associated with spinosad response in *L. decemlineata* exposed to this compound using high-throughput sequencing. Results presented show multiple transcripts of interest that exhibit differential expression in spinosad-treated *L. decemlineata* and provide a preliminary footprint of transcripts affected by this insecticide in this potato pest. Select targets identified in this signature should be further explored in follow-up studies to better characterize their contribution, if any, in the process of spinosad resistance.

**Abstract:**

The Colorado potato beetle *Leptinotarsa decemlineata* is an insect pest that threatens potato crops globally. The primary method to control its damage on potato plants is the use of insecticides, including imidacloprid, chlorantraniliprole and spinosad. However, insecticide resistance has been frequently observed in Colorado potato beetles. The molecular targets and the basis of resistance to imidacloprid and chlorantraniliprole have both been previously quantified. This work was undertaken with the overarching goal of better characterizing the molecular changes associated with spinosad exposure in this insect pest. Next-generation sequencing was conducted to identify transcripts that were differentially expressed between Colorado potato beetles exposed to spinosad versus control insects. Results showed several transcripts that exhibit different expression levels between the two conditions, including ones coding for venom carboxylesterase-6, chitinase 10, juvenile hormone esterase and multidrug resistance-associated protein 4. In addition, several microRNAs, such as miR-12-3p and miR-750-3p, were also modulated in the investigated conditions. Overall, this work reveals a molecular footprint underlying spinosad response in Colorado potato beetles and provides novel leads that could be targeted as part of RNAi-based approaches to control this insect pest.

## 1. Introduction

The Colorado potato beetle *Leptinotarsa decemlineata* (Say) (Coleoptera: Chrysomelidae) can significantly harm potato crops and is an insect pest of substantial economic importance for the agricultural industry worldwide [1]. Complete yield loss of potato crops was reported when defoliation was greater than 75%, underscoring the damage that this insect can cause to potato fields [2]. Several approaches, including crop rotation [3,4], biopesticides [5], and transgenic plants [6] have been leveraged to control this insect with various levels of success. The use of insecticides remains a primary means by which Colorado potato beetles are targeted even though greater resistance against select compounds has been observed in recent years. Decreased susceptibility against the neonicotinoids thiamethoxam and clothianidin was reported in populations of Colorado potato beetles collected in Canada [7]. Resistance to the neonicotinoid imidacloprid has also been reported in several studies [8,9]. Work on a population of Colorado potato beetles collected in Long Island notably displayed a 309-fold increase in imidacloprid resistance when compared with control insects [10]. Interestingly, imidacloprid-resistant insects sampled from the same location displayed low levels of resistance towards the compound spinosad.

Spinosad, an insecticide obtained following fermentation of the actinomycete *Saccharopolyspora spinosa* and comprising spinosyns A and D [11] has raised interest for its use in controlling Colorado potato beetles, even though the complete molecular signature linked with its use in this insect has yet to be determined. Spinosad is particularly of interest to organic growers and has been registered to control several insect pests in multiple countries on certified organic horticultural crops. However, resistance to spinosad has been documented in an increasing number of insects. Spinosad resistance has been found for the tomato borer *Tuta absoluta* (Lepidoptera: Gelechiidae) [12], the diamondback moth *Plutella xylostella* (Lepidoptera: Plutellidae) [13], the leafworm moth *Spodoptera litura* (Lepidoptera: Noctuidae) [14] and the fall armyworm *Spodoptera frugiperda* (Lepidoptera: Noctuidae) [15] to name a few. Pioneering work has shown that mutations in subunit α6 of the nicotinic acetylcholine receptor (nAChR) were associated with increased spinosad resistance in *Drosophila melanogaster* (Diptera: Drosophilidae) [16]. Work undertaken in *P*. *xylostella* exhibiting different degrees of response to spinosad showed substantial levels of γ-aminobutyric acid receptor (GABAR) transcripts in the spinosad-resistant strain when compared with a sensitive counterpart [17]. Spinosad treatment of *S. frugiperda* Sf9 cells was associated with induction of autophagy as well as programmed cell death [18,19]. High-throughput approaches have also been leveraged to better understand the molecular changes underlying spinosad resistance in insects. For example, whole transcriptome analysis conducted for strains of olive fly *Bactrocera oleae* (Diptera: Tephritidae) that were sensitive versus resistant to spinosad revealed differentially expressed genes associated with differential energy requirements in the resistant strain [20]. While these studies provide evidence of the molecular impact resulting from spinosad resistance in diverse pests, information is sparse regarding the effect of this compound on the Colorado potato beetle.

In this study, we undertook a transcriptomics-based approach to identify differentially expressed transcripts related to spinosad response in *L. decemlineata*. We quantified differentially expressed mRNAs and miRNAs in Colorado potato beetles treated with spinosad as compared to controls. We present a rationale for the modulation of these targets in the context of insecticide responses in Colorado potato beetles.

## 2. Materials and Methods

### 2.1. Insects

*L. decemlineata* adults were obtained from the Fredericton Research and Development Centre colony in Fredericton (NB, Canada) in July 2018. Insects were brought back to Université de Moncton (Moncton, NB, Canada) in plastic containers supplemented with potato (*Solanum tuberosum* var. Shepody) leaves. Upon arrival, insects were housed in a cage with potato plants until use in experiments. A group of insects was acclimated in an incubator (Thermo Fisher Scientific, Waltham, MA, USA) set at 25 °C for 5 days under 16 L:8 D cycles and containing potato plants. A volume of 0.5 µL spinosad (Sigma #33706, dissolved in acetone, Sigma-Aldrich, St. Louis, MO, USA) at a concentration of 1 µg/µL was pipetted topically on the abdomen of 15 beetles. An equal volume of acetone was applied to 15 beetles used as controls. Insects were placed back into the incubator for 4 h. All insects displayed signs of activity after the incubation period and prior to storage. It is relevant to point out that more insects (15) were treated and stored for each condition than the number of insects required to generate the results presented in this specific study. Additional insects were stored for use in follow-up work relevant to spinosad response in *L. decemlineata*. Parallel exposure tests were also performed on groups of five *L. decemlineata* exposed to doses of 0.5, 1.0, 2.0, 5.0, 10.0, 20.0 and 40.0 µg of spinosad. Activity impairment and insect lethality was observed after 24 h in ≥40% of insects at the five highest doses, and the lowest dose (0.5 µg) was thus selected for this study (Supplementary Materials Table S1). Insect mobility impairment was assessed via moderate agitation of the dish and subsequent evaluation of the ability of the insect to right itself [21]. Insects were sampled by being placed rapidly in liquid nitrogen and stored at −80 °C until use.

### 2.2. RNA Isolation

Small and large RNA fractions from control and spinosad-treated *L. decemlineata* were generated, as previously reported [22], with the miRVana miRNA Isolation Kit (Thermo Fisher Scientific) following manufacturer’s instructions and using two insects as the starting material for each replicate. Briefly, insects were homogenized in lysis/binding buffer using a mechanical homogenizer. Extraction was conducted using an acid-phenol/chloroform solution. Filter cartridges were used to obtain fractions enriched with small RNAs as well as fractions containing large RNAs depleted of small RNAs. A NanoVue Plus Spectrophotometer (Thermo Fisher Scientific) was used to measure RNA concentrations. Small and large RNA isolates were stored at −80 °C until analysis by next-generation sequencing for the spinosad-associated expression of miRNAs and mRNAs, respectively.

### 2.3. Large RNA Library Construction and Sequencing

Large RNA fractions (*n* = 3) generated with two insects per replicate were quantified and RNA integrity was analyzed on an Agilent TapeStation (Agilent, Santa Clara, CA, USA). Samples with RNA integrity numbers above 8 were selected for library preparation. Poly(A) enrichment was performed from 20 µg of total RNA to enrich for mRNA using a Dynabeads mRNA Direct Micro Purification Kit (Thermo Fisher Scientific). An amount of 100 ng of poly(A)-enriched RNA was fragmented with RNase III and purified using the magnetic bead purification module Ion Total RNA-Seq v2 kit (Thermo Fisher Scientific). Size distribution of the fragmented RNA was assessed on the Agilent TapeStation using High Sensitivity RNA ScreenTape. A total of 50 ng of fragmented poly(A)-enriched RNA was used to prepare whole transcriptome libraries using Ion Total RNA-Seq v2 kit (Thermo Fisher Scientific). Poly(A) transcripts were ligated onto Ion Torrent adapters followed by reverse transcription. Resulting cDNA transcripts were purified and barcoded libraries were generated using Ion Xpress RNA barcodes (Thermo Fisher Scientific). Conditions recommended by Ion Torrent were followed throughout the protocol. Yield and size distribution of libraries were analyzed on an Agilent TapeStation using D1000 ScreenTape. Libraries were then equally pooled by pooling 7 pM libraries from each sample, and amplified onto ion sphere particles (ISPs) supplied by the Ion PI Hi-Q OT2 kit (Thermo Fisher Scientific). ISPs enriched with template libraries were loaded onto the Ion PI chip v3 and next-generation sequencing (NGS) was performed on an Ion Proton System (Thermo Fisher Scientific).

Raw poly(A) RNA-Seq reads were processed with Torrent Suite Software v5.10.1, which runs the Torrent Mapping Alignment Program (TMAP) using the *L. decemlineata* assembly genome version Ldec 2.0 from NCBI [23]. The number of genes in the *L. decemlineata* genome predicted in this assembly genome totaled 24,671 gene transcripts and 93,782 predicted exons. Reads were then submitted to a two-step mapping process. The first step consisted of mapping using STAR v.2.5.2b [24], while the unmapped reads were subsequently mapped using Bowtie 2 v2.2.5 [25]. The two aligned files were merged using Picard tools (release 2.5) [26]. Raw counts were extracted with featureCounts v1.4.6-p4 [27]. Gene expression in fragments per kilobase of transcript per million mapped reads (FPKM) values was subsequently calculated with Cufflinks v2.2.1 [28], an approach supported for single-end reads assessment on this sequencing platform and exemplified elsewhere [29,30]. To test for statistical differences in differentially expressed genes between the control and treatment conditions, unpaired Student’s *t*-tests were performed in the R statistical environment v3.4.1 [31] after removing genes exhibiting low expression (FPKM < 1 in two samples for both conditions).

### 2.4. Small RNA Library Construction and Sequencing

Small RNA fractions quality (*n* = 4), generated with two insects per replicate, was assessed on an Agilent TapeStation using a standard RNA ScreenTape assay. Small RNA libraries were prepared from 250 ng of enriched small RNA using the RNA-Seq v2 kit (Thermo Fisher Scientific). Small RNA transcripts were ligated to Ion Torrent adapters followed by reverse transcription (Ion Total RNA-seq v2 kit, Thermo Fisher Scientific). Resulting cDNA products were next subjected to double size selection. This step removed the majority of larger cDNA fragments and enriched the cDNA fragments from small RNA transcripts. Barcoded libraries were generated from the enriched cDNA fragments using Ion Xpress RNA barcodes (Thermo Fisher Scientific). Ion Torrent recommended conditions were followed throughout the protocol. Quality of the libraries was analyzed using a TaqMan library quantification assay (Ion Library TaqMan Quantitation Kit, Thermo Fisher Scientific). Barcoded libraries were equally pooled and amplified onto ISPs supplied with the Ion PI Hi-Q OT2 kit (Thermo Fisher Scientific). ISPs enriched with template libraries were loaded onto Ion PI chip v3 and sequenced as above.

Small RNA-Seq raw reads were processed with Torrent Suite Software v5.10.1, which runs the Torrent Mapping Alignment Program (TMAP) using the *L. decemlineata* genome mentioned above. Removal of the adapters and selection of reads 15 to 30 bp in length were performed using Cutadapt v1.8.1 [32] and Picard tools (release 2.5) [26]. Reads were mapped to miRNA sequences of *L. decemlineata* obtained from the Ldec 2.0 genome [23] using Bowtie 2 v2.2.5 [25], and read counts were obtained with featureCounts v1.4.6-p4 [27]. R statistical environment v3.4.1 and Bioconductor package edgeR v3.18.1 [33] were used to identify differentially expressed miRNAs. Transcripts with fewer than 10 reads in more than half the samples under both conditions were removed. Analysis was performed using the trimmed mean of M-values (TMM) normalization method [34]. Transcriptomics data sets relevant to the current study are accessible through the Sequence Read Archive (SRA) of NCBI under the bioproject identification PRJNA635016.

### 2.5. Synthesis of cDNA

First strand synthesis for mRNA amplification was performed by mixing 1 µg of total RNA—which was obtained from large RNA isolates that were previously RNA isolates (3) used for NGS work or new isolates (2) not used as part of NGS analysis—with 1 µL oligo dT and 1 µL 10 mM dNTPs. Diethyl pyrocarbonate (DEPC)-treated water was next added to a volume of 12 µL. The solution was incubated at 65 °C for 5 min. These reagents were subsequently added to the mixture: 4 µL 5× First Strand Buffer, 2 µL 0.1 M DTT and 1.5 µL DEPC-treated water. This solution was incubated at 37 °C for 2 min, then 0.5 µL of M-MLV Reverse Transcriptase (RT) was added. Final incubation steps at 37 °C for 50 min and 70 °C for 15 min were ultimately performed.

### 2.6. qRT-PCR Amplification of Spinosad-Associated Transcripts Identified via NGS

Primers for amplification and quantification of transcripts identified via NGS were conceived based on *L. decemlineata* sequences for each transcript. Primers are presented in Table 1. Initial reactions contained 5 µL of diluted cDNA template (10^−1^), 1 µL 25 µM forward primer, 1 µL 25 µM reverse primer, 5.5 µL DEPC-treated water and 12.5 µL 2X Taq FroggaMix (FroggaBio, Concord, ON, Canada). Amplification protocol consisted of a denaturing step at 95 °C for 5 min, followed by 40 cycles at 95 °C for 15 s, at a temperature gradient between 50 and 65 °C for 30 s and at 72 °C for 45 s. Amplified products were resolved on a 2% agarose gel and sequenced at the Université Laval sequencing platform (Quebec City, QC, Canada). Targets of interest were also amplified via qRT-PCR at different annealing temperatures and on serial cDNA dilutions to measure primer set efficiencies. Reactions conducted to quantify transcript levels consisted of 2.5 µL of cDNA template (10^−1^), 0.5 µL DEPC-treated water, 1 µL 5 µM forward primer, 1 µL 5 µM reverse primer and 5 µL of iTaq Universal SYBR Green Supermix (Bio-Rad, Hercules, CA, USA). The amplification protocol comprised an initial step at 95 °C for 3 min, followed by 40 cycles at 95 °C for 15 s and at the identified optimal annealing temperature for 30 s. Transcript levels of α-tubulin were used as reference and were amplified as above using 55 °C as the annealing temperature. Significant differences between transcript target expression measured in the control versus spinosad-exposed insects were assessed with an unpaired Student’s *t*-test. Relative normalized transcript expression and a statistical analysis of the data were conducted with CFX Maestro software v1.1 (Bio-Rad).

### 2.7. Functional Assessment of Spinosad-Modulated mRNAs

Functional annotation of the most differentially expressed mRNA transcripts in spinosad-exposed *L. decemlineata*, as determined by next-generation sequencing was performed using Blast2GO Basic v5.2.5 [35]. Up- and down-regulated *L. decemlineata* transcript gene sequences were mapped using the Blast Diptera database (taxa: 7147) to identify homologous dipteran transcripts to use for functional classification with Blast2GO. Dipteran genes obtained with e-values below the cut-off of 1.0 E-3 were used for subsequent functional annotation.

## 3. Results

### 3.1. mRNA Expression in Spinosad-Exposed L. decemlineata by NGS

**Figure 1:**
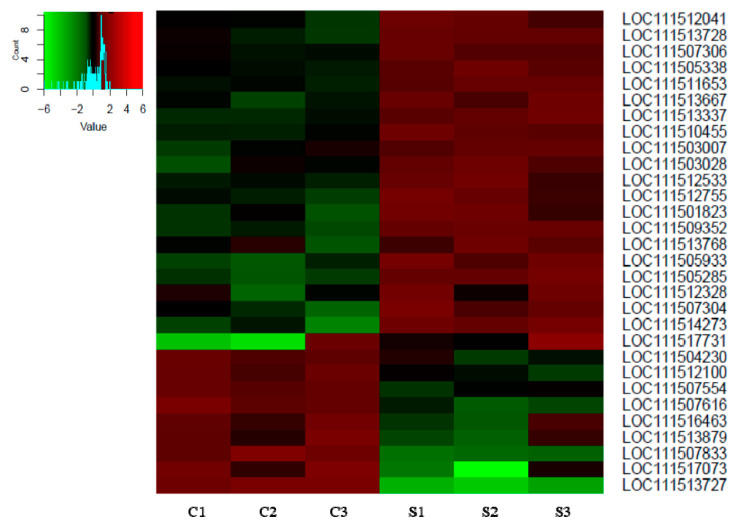
Heatmap showing the most differentially expressed transcripts in control (C) insects versus insects treated with spinosad (S) as measured by NGS. Accession numbers for transcripts that showed absolute log2 fold-changes greater than 1.0 and *p* values lower than 0.05.

Next-generation sequencing revealed 182,009,095 reads among all samples. Filtering was performed to discard low-quality reads and generated an average of 27,398,426 and 21,001,758 mapped reads for control and spinosad-treated insects, respectively. Reads were mapped to an average of 13,281 genes among all samples. Transcripts with absolute log2 fold-changes greater than 1.0 and *p* values below 0.05 were further identified. Up-regulation in spinosad-treated *L. decemlineata* was measured for 21 transcripts, and down-regulation was monitored for nine transcripts. Results are presented in Figure 1 and Table 2. Transcripts showing the greatest up-regulation in insects treated with spinosad as determined by next-generation sequencing were peritrophin-1-like (LOC111514273), polygalacturonase/glycoside hydrolase family 28 (LOC111505285), cathepsin B-like (LOC111505933), lysosomal alpha-mannosidase-like (LOC111509352) and venom carboxylesterase-6-like (LOC111507304). Transcripts exhibiting the most reduced levels in spinosad-exposed insects when compared with control insects were cathepsin L proteinase (LOC111513727), voltage-dependent calcium channel type A subunit alpha-1-like (LOC111507833), putative nuclease HARBI1 (LOC111517073), senecionine N-oxygenase-like (LOC111507616) and lipase 3-like (LOC111513879).

Transcripts coding for targets with proposed relevance to insecticide responses in *L. decemlineata*, including cytochrome P450s [36] and cuticular proteins [37], were also identified as above using less stringent cut-offs. Transcripts displaying absolute log2 fold-changes above 0.25 and *p* values below 0.1 were obtained from NGS data. Results are shown in Table 3. Transcripts showing the greatest up-regulation in spinosad-exposed insects versus control insects consisted of the ones coding for cytochrome P450 9e2 (LOC111506250) and cytochrome P450 6k1 (LOC111510879), while transcripts exhibiting the strongest down-regulation under the same conditions consisted of a mitochondrial cytochrome P450 301a1 (LOC111516463) and cytochrome P450 4c1 (LOC111509981).

### 3.2. qRT-PCR-Based Expression of Transcripts Modulated by Spinosad in L. decemlineata as Determined by NGS

**Figure 2:**
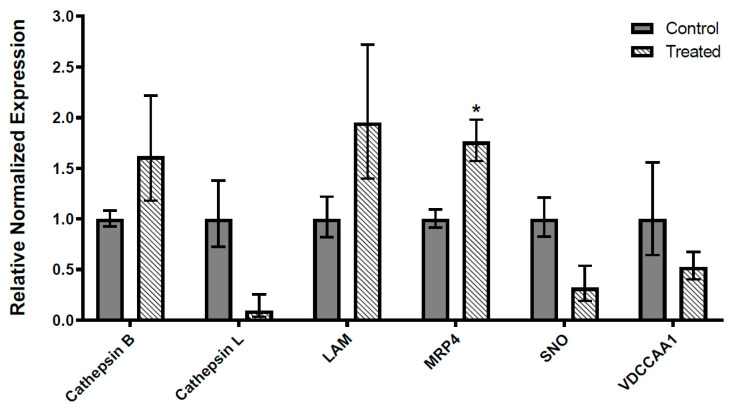
Expression of transcript levels in spinosad-treated versus control insects. Data presented correspond to mean standardized transcript levels (mean ± SEM, *n* = 5). Asterisk identifies results that significantly differ from control samples (* *p* < 0.05). Abbreviations: Cathepsin L-like proteinase (Cathepsin L), lysosomal α-mannosidase (LAM), multidrug resistance-associated protein 4 (MRP4), senecionine N-oxygenase (SNO) and voltage-dependent calcium channel type A subunit alpha-1 (VDCCAA1).

Expression of six targets with varying levels following NGS was measured by qRT-PCR in control and insecticide-exposed insects. Results are shown in Figure 2. Transcript levels of cathepsin B (LOC111505933), lysosomal alpha-mannosidase (LOC111509352) and multidrug resistance-associated protein 4 (LOC111503007) were elevated in spinosad-treated insects to relative normalized expression values that were 1.62-, 1.95- and 1.76-fold higher than the ones measured in control insects, respectively. Transcript levels of cathepsin L proteinase (LOC111513727), senecionine N-oxygenase (LOC111507616) and voltage-dependent calcium channel type A subunit alpha-1 (LOC111507833) displayed reduced expression in spinosad-exposed insects to relative normalized expression values that were 0.09-, 0.32- and 0.52-fold higher than the ones observed in untreated insects, respectively. Changes observed by qRT-PCR displayed similar trends in expression to the ones measured by NGS for all transcripts, although most reported changes were not statistically significant.

### 3.3. miRNA Expression in Control and Spinosad-Exposed Insects via NGS

High-throughput sequencing revealed miRNAs with elevated levels in *L. decemlineata*, including miRNA miR-8-3p (82,033 reads in control and 48,570 reads in spinosad-treated insects), miR-14-5p (57,408 reads in control and 69,918 reads in spinosad-treated insects) and miR-317-5p (49,768 reads in control and 34,526 reads in spinosad-treated insects). A list of the most strongly expressed miRNAs in *L. decemlineata* is shown in Table 4. Furthermore, miRNA transcripts with absolute log2 fold-changes above 0.5 and *p* values below 0.05 were identified. Ratios of miRNA expression in spinosad-treated versus control *L. decemlineata* revealed differential expression of 14 miRNAs. A total of nine up-regulated miRNAs, including miR-750-3p, miR-2796-5p and miR-3791-5p, as well as five down-regulated, such as miR-8-3p, miR-9a-5p and miR-12-3p, were identified. MiRNAs modulated in spinosad-exposed insects are presented in Table 5.

### 3.4. Functional Classification of Differentially Expressed mRNA Transcripts Following Spinosad Treatment

Functional annotation of spinosad-modulated transcripts as determined by next-generation sequencing was performed. Biological processes were obtained for select transcripts that displayed substantial increased or decreased expression in *L. decemlineata* exposed to spinosad. Identified transcripts were associated, using Blast2GO, with GO terms that included primary metabolic process (GO:0044238), proteolysis (GO:0006508), nucleoside transmembrane transport (GO:1901642), response to hypoxia (GO:0001666), oxidation-reduction process (GO:0055114), lipid metabolic process (GO:0006629) and ion transport (GO:0006811), to name a few. A complete list of the processes identified is presented in Table 6.

## 4. Discussion

Successful management of insecticide resistance in insect pests that threaten agricultural production requires a better understanding of the transcriptional changes that occur in response to exposure to a given chemical. Multiple reports have leveraged transcriptomics-based approaches in order to highlight the molecular changes observed in *L. decemlineata* exposed to diverse compounds including chlorothalonil and imidacloprid [38]. However, similar studies are lacking with respect to spinosad response in *L. decemlineata*, despite its increased usage and need by organic producers. This work thus aimed to better characterize the modulated transcripts underlying spinosad response in this insect pest.

Multiple transcripts were identified in this study as differentially expressed in spinosad-exposed Colorado potato beetles when compared with control insects using high-throughput sequencing. Varying levels of select transcripts with functions linked to insecticide resistance were observed. For example, elevated transcript levels of venom carboxylesterase-6 (LOC111507304), a carboxylesterase (CBE), were measured in spinosad-treated *L. decemlineata*. Multiple reports have showcased the involvement of CBEs in the resistance of insects towards different chemicals including organophosphates, carbamates and pyrethroids [39]. Assessment of CBE activity in the diamondback moth *P. xylostella* demonstrated a positive correlation with several insecticides including spinosad [40]. Members of a CBE and cholinesterase superfamily have also been previously investigated in *L. decemlineata*, and it was concluded that their expression could be influenced following insect treatment with fipronil or cyhalotrin [41]. Interestingly, mutations in a carboxylesterase gene of the house fly *Musca domestica* (Diptera: Muscidae) are potentially correlated with spinosad resistance [42]. Besides CBE, up-regulation of a transcript coding for multidrug resistance-associated protein 4 (LOC111503007) was also observed following spinosad treatment in the current work. Modulation of multidrug resistance-associated proteins with the primary function of transporting a broad array of compounds (e.g., xenobiotics) across cellular membranes [43] has been observed in several insects exposed to insecticides. Exposition of the bird cherry-oat aphid *Rhopalosiphum padi* (Hemiptera: Aphididae) to imidacloprid or chlorpyrifos was associated with elevated transcript levels of ABCC1, coding for the multidrug resistance-associated protein 1 (MRP1) [44]. Furthermore, the same study reported higher levels of this target in insect strains that were resistant to these chemicals. It is interesting to note that several genes coding for ATP-binding cassette transporters displayed increased transcript levels in imidacloprid-resistant *L. decemlineata* [45]. Furthermore, significant elevation of ATP-binding cassette subfamily G transporter transcript levels was observed in a commercially managed population of *L. decemlineata* that was imidacloprid-resistant when compared with an organically managed susceptible counterpart [46], which also supports the relevance of such transporters in insecticide resistance with respect to this insect pest. Functional annotation performed in the current work highlighted processes associated with multidrug resistance-associated protein 4, including response to oxidative stress. This aligns with previous work showing that spinosad can induce oxidative stress in various models including Sf9 cells [47] and the Nile tilapia *Oreochromis niloticus* [48]. Future work is nevertheless warranted to build on the functional insights obtained here regarding the identified transcripts with potential relevance to spinosad. Subsequent in-depth functional enrichment analysis and a closer investigation of possible gene orthologues existing in other species with better defined functions would suit this objective. Overall, the data presented here reveal that spinosad treatment in Colorado potato beetles was linked with differential expression of multiple transcripts associated with insecticide responses.

Spinosad exposure also led to differential expression in *L. decemlineata* of transcripts underlying insect development. Chitinase 10 (LOC111512533) was up-regulated in insects treated with this compound. Chitinases can degrade the polysaccharide chitin, a key exoskeletal component in insects, and their underlying activity is notably important in insect development and molting [49]. Previous studies have explored the potential contribution of chitinases towards insecticide responses. For example, treatment of mosquito *Culex pipiens* (Diptera: Culicidae) larvae with the chemical lambda-cyhalotrin has been associated with elevated chitinase activity [50], while treatment of *D. melanogaster* with the tetranortriterpenoid insecticide azadirachtin has been associated with reduction in chitinase activity [51]. It is interesting to note that in our study, peritrophin-1 (LOC111514273) was strongly up-regulated in *L. decemlineata* treated with spinosad. Peritrophins are chitin-binding proteins and key components of the peritrophic membrane in the insect gut [52]. Reduction of chitin and peritrophins using an approach based on double-stranded RNA (dsRNA) has resulted in increased sensitivity to imidacloprid in the eastern subterranean termite *Reticulitermes flavipes* (Isoptera: Rhinotermitidae) [53]. Besides chitinase 10 and peritrophin-1, elevated transcript levels of juvenile hormone esterase (LOC111512328) were also detected in this study. Exposure of *P. xylostella* to pyriproxyfen has been correlated with elevated transcript levels and the activity of juvenile hormone esterase [54]. In addition, treatment of diamondback moth larvae with 3-octylthio-1,1,1-trifluoropropan-2-one (OTFP), a trifluoromethyl ketone that displayed inhibitory activity against juvenile hormone esterase, was associated with differential responses to various chemicals such as diafenthiuron and indoxacarb [55]. Collectively, these changes highlight key modulation in Colorado potato beetles of select targets involved in insect development following spinosad exposure.

Data presented here also showed differential expression of several miRNAs in Colorado potato beetles exposed to spinosad when compared with control insects. Since the pioneering report that identified 460 putative miRNAs in *L. decemlineata* [56], several studies have reported miRNA modulation in Colorado potato beetles exposed to different insecticides. A next-generation sequencing-based approach revealed the modulation of 33 miRNAs, such as miR-100 and miR-989, in *L. decemlineata* treated with imidacloprid [57]. The former was notably shown to target the cytochrome P450 CYP6CY3, which had been linked with neonicotinoid resistance [58,59]. Numerous specific differentially expressed cytochrome P450s, part of the CYP450 gene superfamily from which multiple members have been investigated for their underlying role in insecticide resistance in *L. decemlineata* [60,61], were identified via high-throughput sequencing in the current work, supporting further investigation of the miRNA-mediated regulation of cytochrome P450s following spinosad exposure. A different signature of miRNAs displaying up-regulation of miR-1-3p and miR-305-5p was observed in *L. decemlineata* exposed to chlorantraniliprole [22]. Recent work also reported the potential involvement of miR-9a-5p and miR-965-5p in hexanoic acid response in *L. decemlineata* [62]. The current study highlighted a signature of different miRNAs that were responsive to spinosad, including miR-9a-5p, miR-12-3p and miR-750-3p, which further highlights the variations in miRNA footprints elicited by specific insecticides in *L. decemlineata*. Subsequent work aimed at better clarifying the targets regulated by these miRNAs is warranted to assess the potential relevance of these leads in spinosad response in this insect pest. It is also worth pointing out that follow-up investigations using longer spinosad exposures would contribute to a more in-depth characterization of molecular changes, both short- and mid-term, associated with spinosad response in *L. decemlineata*. Treatment protocols using higher doses of spinosad, combined with increased stringency of the thresholds used for NGS data analysis, could also provide a more thorough and rigorous overview of the transcriptional variations linked with response to this chemical and build on the data reported in this study given the limited number of transcripts presented.

## 5. Conclusions

Given the accumulating reports of spinosad resistance in select populations of Colorado potato beetles [63] and the long-term objective of better characterizing the changes in gene expression potentially affected by spinosad, the current study investigated the molecular changes linked to spinosad response in Colorado potato beetles using a high-throughput sequencing approach. A signature of mRNAs coding for diverse targets with relevance to spinosad response were identified, including select chitinases, multidrug resistance-associated protein and juvenile hormone esterase. Expression levels of select transcripts identified using the NGS approach were subsequently quantified via qRT-PCR in an expanded number of replicates. Comparable trends in expression status, albeit not all statistically significant, were observed following NGS- versus qRT-PCR-based quantification. In addition, spinosad-associated differential expression of miRNAs, including miR-12-3p and miR-750-3p, was also observed for the first time in *L. decemlineata* exposed to spinosad. Overall, this work showed signatures of transcripts that responded to spinosad in *L. decemlineata* and further revealed potential targets to consider when designing novel approaches aimed at limiting the damages this insect can cause to potato fields.

## Figures and Tables

**Table 1:** Primers for the qRT-PCR-based quantification of spinosad-associated transcripts, as measured by next-generation sequencing (NGS). Abbreviations: Forward (Fwd) and reverse (Rev) primers.

Primer	Sequence		Efficiency	Temperature
Cathepsin B	Fwd Rev	5′-TTCGATGCACGAGAAAATTG-3′	100.3%	57.7 °C
		5′-CTCATGACGGAAGTTGCTGA-3′		
Cathepsin L-like proteinase	Fwd Rev	5′-AACGCAGTAGGTACCGTTGG-3′	101.3%	57.1 °C
		5′-CAGCCAAAACACCATGATTG-3′		
Lysosomal alpha-mannosidase	Fwd Rev	5′-GAAATTGGTTCAGCCTTGGA-3′	99.8%	58.1 °C
		5′-ATAGCTGCGCGTCTTCATTT-3′		
Multidrug resistance-associated protein 4	Fwd Rev	5′-TCCAATGTGCGAAACAACAT-3′	102.1%	57.1 °C
		5′-GGCAAAGGGAAATTGTTTGA-3′		
Senecionine N-oxygenase	Fwd Rev	5′-TGTTGTGGTTTCGACGTCAT-3′	103.6%	58.1 °C
		5′-TACGTTGAACGGAACACCAA-3′		
Voltage-dependent calcium channel type A subunit alpha-1	Fwd Rev	5′-AGCATAGCCATCCATCCTTG-3′	100.4%	63.2 °C
		5′-GAGTCTGCGGTGTAGCATGA-3′		
α-tubulin	Fwd Rev	5′-GAGTTCCAGACCAACTTGGT-3′	107.9%	52.6 °C
		5′-GCCATGTACTTGCCGTGACG-3′		

**Table 2:** Modulation of 30 mRNA transcripts in spinosad-treated *L. decemlineata*. Accession numbers, name of transcripts and changes in expression levels for spinosad-modulated transcripts identified by NGS.

Target Accession Number	Target Name	Log2 Fold-Change	*p*-Value
		Spinosad/Control	
LOC111514273	Peritrophin-1-like	2.08	0.046
LOC111505285	Polygalacturonase-like|glycoside hydrolase family 28	1.88	0.020
LOC111505933	Cathepsin B-like	1.83	0.019
LOC111509352	Lysosomal alpha-mannosidase-like	1.65	0.003
LOC111507304	Venom carboxylesterase-6-like	1.60	0.036
LOC111501823	Lysosomal alpha-mannosidase-like	1.45	0.041
LOC111512755	Cathepsin L-like proteinase	1.45	0.039
LOC111513337	GILT-like protein 1	1.43	0.023
LOC111517731	Uncharacterized protein LOC111517731	1.39	0.032
LOC111513667	Tetratricopeptide repeat protein 39B-like	1.38	0.016
LOC111512533	Probable chitinase 10	1.30	0.048
LOC111510455	Equilibrative nucleoside transporter 3-like	1.28	0.041
LOC111511653	Uncharacterized protein LOC111511653	1.24	0.012
LOC111512328	Juvenile hormone esterase-like	1.23	0.024
LOC111513728	Cathepsin L-like proteinase	1.20	0.006
LOC111503028	Neutral alpha-glucosidase C-like	1.16	0.028
LOC111512041	Venom carboxylesterase-6-like	1.16	0.008
LOC111505338	Uncharacterized protein LOC111505338	1.13	0.035
LOC111503007	Multidrug resistance-associated protein 4-like	1.02	0.007
LOC111513768	Juvenile hormone acid O-methyltransferase-like	1.02	0.044
LOC111507306	Venom carboxylesterase-6-like	1.01	0.006
LOC111504230	Uncharacterized protein LOC111504230	−1.04	0.001
LOC111516463	Probable cytochrome P450 301a1, mitochondrial|cytochrome P450 12h2	−1.07	0.008
LOC111507554	Uncharacterized protein LOC111507554	−1.10	0.033
LOC111512100	Neuropeptide-like protein 31	−1.20	0.050
LOC111513879	Lipase 3-like	−1.32	0.020
LOC111507616	Senecionine N-oxygenase-like	−1.86	0.025
LOC111517073	Putative nuclease HARBI1	−2.24	0.020
LOC111507833	Voltage-dependent calcium channel type A subunit alpha-1-like	−2.83	0.033
LOC111513727	Cathepsin L-like proteinase	−5.13	0.005

**Table 3:** Differential expression of select transcripts with putative relevance to insecticide responses in *L. decemlineata* treated with spinosad as measured by high-throughput sequencing.

Target Accession Number	Target Name	Log2 Fold-Change	*p*-Value
		Spinosad/Control	
LOC111506250	Cytochrome P450 9e2-like	1.41	0.051
LOC111510879	Cytochrome P450 6k1-like	0.92	0.069
LOC111517755	Probable cytochrome P450 6a23/cytochrome P450	0.91	0.026
LOC111517753	Probable cytochrome P450 6a23/cytochrome P450 6bj3	0.57	0.064
LOC111503064	Cytochrome P450 4g15	0.42	0.071
LOC111505596	Endocuticle structural glycoprotein SgAbd-4-like/putative cuticle protein CP6	0.39	0.067
LOC111505534	NADPH-cytochrome P450 reductase	0.33	0.041
LOC111503441	Cytochrome P450 4c1-like	0.31	0.022
LOC111517496	Endocuticle structural glycoprotein SgAbd-8-like	−0.30	0.095
LOC111505902	Cytochrome P450 CYP12a2-like/cytochrome P450 353a2	−0.49	0.059
LOC111509981	Cytochrome P450 4c1-like	−1.06	0.076
LOC111516463	Probable cytochrome P450 301a1, mitochondrial/cytochrome P450 12h2	−1.07	0.008

**Table 4:** Strongly expressed miRNAs in *L. decemlineata* as determined by high-throughput sequencing. Results shown are average normalized expressions for all samples.

miRNAs	Average Normalized Expression
Lde-miR-8-3p	65,301.25
Lde-miR-14-5p	63,662.96
Lde-miR-317-5p	42,146.90
Lde-miR-1-3p	24,099.48
Lde-bantam-3p	14,830.90
Lde-miR-281-5p	12,820.94
Lde-miR-1175-3p	9564.56
Lde-miR-34-3p	8436.31
Lde-miR-12-3p	6915.56
Lde-miR-13b-3p	6217.84

**Table 5:** Spinosad-modulated miRNAs in *L. decemlineata* as determined by NGS. Presented miRNA transcripts displayed absolute log2 fold-changes above 0.5 and *p* values below 0.05.

miRNAs	Log2 Fold-Change	*p*-Value
	Spinosad/Control	
Lde-miR-750-3p	1.72	0.0034
Lde-miR-2796-5p	1.68	0.0190
Lde-miR-3791-5p	1.50	0.0333
Lde-miR-750-5p	1.07	0.0003
Lde-bantam-5p	0.99	0.0399
Lde-miR-1175-3p	0.92	0.0087
Lde-miR-184-3p	0.87	0.0045
Lde-miR-281-5p	0.67	0.0296
Lde-miR-125-3p	0.64	0.0263
Lde-miR-9e-3-5p	−0.69	0.0071
Lde-let-7-3p	−0.76	0.0267
Lde-miR-8-3p	−0.76	0.0109
Lde-miR-9a-5p	−0.97	0.0001
Lde-miR-12-3p	−1.01	0.0057

**Table 6:** Processes associated with select spinosad-modulated mRNA transcripts in *L. decemlineata*. *L. decemlineata* accession numbers and corresponding targets obtained from the dipteran database are shown.

Target Accession Number	Target Name	GO IDs	GO Terms
LOC111501823	Lysosomal alpha-mannosidase	P:GO:0044238; P:GO:0071704	P:primary metabolic process; P:organic substance metabolic process
LOC111512533	Uncharacterized protein LOC101463243 isoform X2	P:GO:0006508; P:GO:0006897	P:proteolysis; P:endocytosis
LOC111510455	Equilibrative nucleoside transporter 1	P:GO:1901642	P:nucleoside transmembrane transport
LOC111503007	Probable multidrug resistance-associated protein lethal(2)03659	P:GO:0001666; P:GO:0006979; P:GO:0008340; P:GO:0034059; P:GO:0048190	P:response to hypoxia; P:response to oxidative stress; P:determination of adult lifespan; P:response to anoxia; P:wing disc dorsal/ventral pattern formation
LOC111516463	Probable cytochrome P450 301a1, mitochondrial	P:GO:0055114; P:GO:0007490	P:oxidation-reduction process; P:tergite morphogenesis
LOC111513879	Lipase 1	P:GO:0006629; P:GO:0016042	P:lipid metabolic process; P:lipid catabolic process
LOC111507833	Voltage-dependent calcium channel type A subunit alpha-1 isoform X14	P:GO:0006811	P:ion transport

## Supplementary Materials

The following are available online at https://www.mdpi.com/2075-4450/11/11/820/s1, Table S1: Impact of spinosad on *L. decemlineata*. Mortality/impairment rates of insects exposed to varying doses of spinosad are shown.
