# Supplementary figures and images for: Transcriptomics-Based Approach Identifies Spinosad-Associated Targets in the Colorado Potato Beetle, Leptinotarsa decemlineata
Source: Insects. 2020 Nov 21;11(11):820. doi: 10.3390/insects11110820 (PMC7700309; doi:10.3390/insects11110820)

## Slide 1
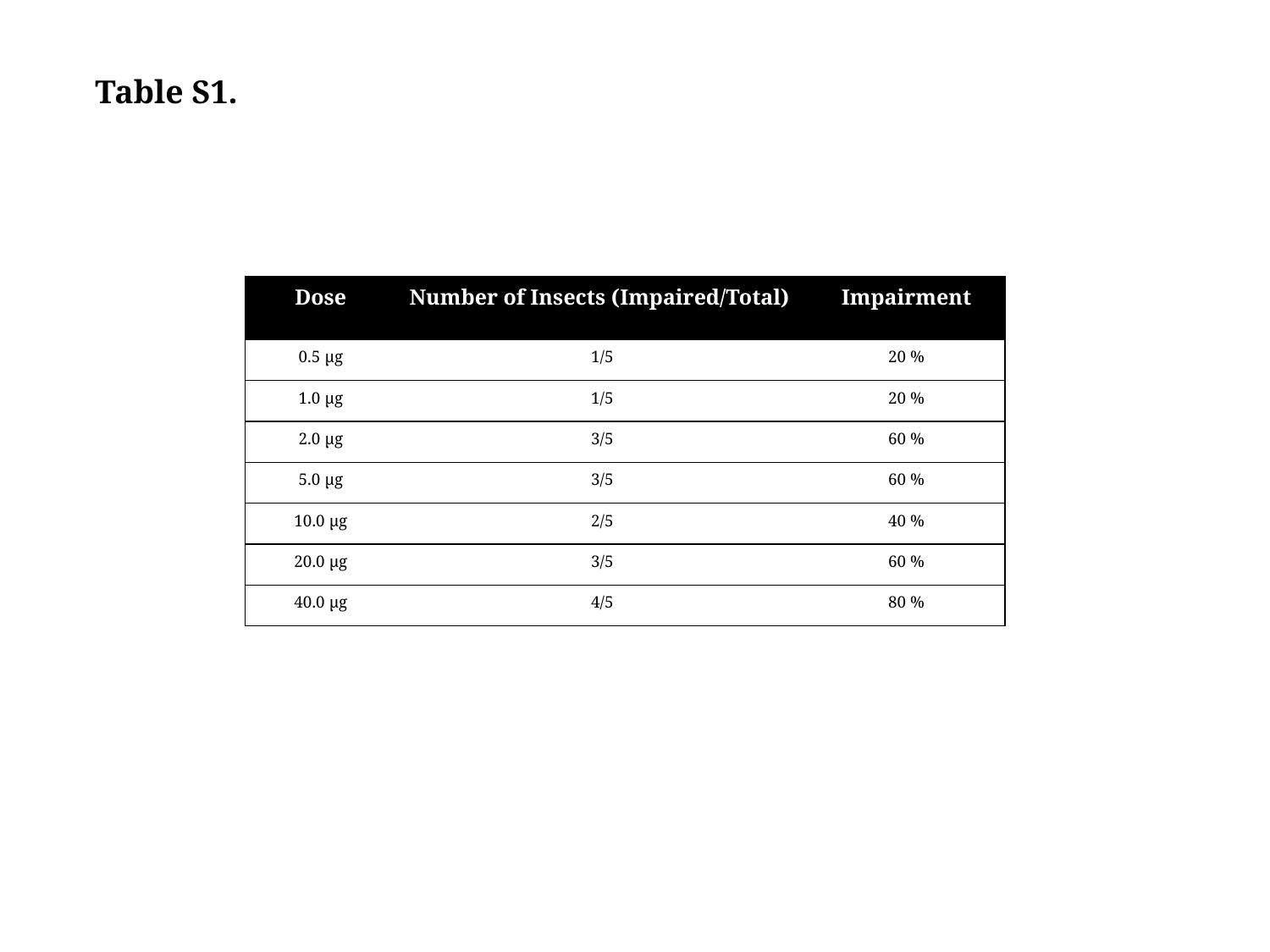

Table S1.
| Dose | Number of Insects (Impaired/Total) | Impairment |
| --- | --- | --- |
| 0.5 µg | 1/5 | 20 % |
| 1.0 µg | 1/5 | 20 % |
| 2.0 µg | 3/5 | 60 % |
| 5.0 µg | 3/5 | 60 % |
| 10.0 µg | 2/5 | 40 % |
| 20.0 µg | 3/5 | 60 % |
| 40.0 µg | 4/5 | 80 % |

Supplement: Supplementary file 1 [file insects-11-00820-s001.zip › insects-1001903.pptx]
